# A phase I trial of autologous RAK cell immunotherapy in metastatic renal cell carcinoma

**DOI:** 10.1007/s00262-024-03680-y

**Published:** 2024-04-20

**Authors:** Jing Xu, Wen Zhang, Jinlian Tong, Caixia Liu, Qiaohui Zhang, Liren Cao, Jiangyong Yu, Aiping Zhou, Jie Ma

**Affiliations:** 1 Center of Biotherapy, Beijing Hospital, National Center of Gerontology, Institute of Geriatric Medicine, Chinese Academy of Medical Sciences, Beijing, 100730 China; 2 Department of Medical Oncology, National Cancer Center/National Clinical Research Center for Cancer/Cancer Hospital, Chinese Academy of Medical Sciences, Peking Union Medical College, Beijing, 100021 People’s Republic of China; 3 Clinical Department, Everbright Cell Medical Biotech Inc., Beijing, 100061 People’s Republic of China; 4 Department of Medical Oncology, Beijing Hospital, National Center of Gerontology, Institute of Geriatric Medicine, Chinese Academy of Medical Sciences, Beijing, 100730 China

**Keywords:** Autologous RAK cell, Immunotherapy, Metastatic renal cell carcinoma, Safety, Efficacy

## Abstract

**Background:**

Treatment of metastatic renal cell carcinoma (mRCC) remains a challenge worldwide. Here, we introduced a phase I trial of autologous RAK cell therapy in patients with mRCC whose cancers progressed after prior systemic therapy. Although RAK cells have been used in clinic for many years, there has been no dose-escalation study to demonstrate its safety and efficacy.

**Methods:**

We conducted a phase I trial with a 3 + 3 dose-escalation design to investigate the dose-related safety and efficacy of RAK cells in patients with mRCC whose cancers have failed to response to systemic therapy (ChiCTR1900021334).

**Results:**

Autologous RAK cells, primarily composed of CD8^+^ T and NKT cells, were infused intravenously to patients at a dose of 5 × 10^9^, 1 × 10^10^ or 1.5 × 10^10^ cells every 28 days per cycle. Our study demonstrated general safety of RAK cells in a total of 12 patients. Four patients (33.3%) showed tumor shrinkage, two of them achieved durable partial responses. Peripheral blood analysis showed a significant increase in absolute counts of CD3^+^ and CD8^+^ T cells after infusion, with a greater fold change observed in naive CD8^+^ T cells (CD8^+^CD45RA^+^). Higher peak values of IL-2 and IFN-*γ* were observed in responders after RAK infusion.

**Conclusion:**

This study suggests that autologous RAK cell immunotherapy is safe and has clinical activity in previously treated mRCC patients. The improvement in peripheral blood immune profiling after RAK cell infusion highlights its potential as a cancer treatment. Further investigation is necessary to understand its clinical utility.

**Supplementary Information:**

The online version contains supplementary material available at 10.1007/s00262-024-03680-y.

## Introduction

Renal cell carcinoma (RCC) is a prevalent genitourinary malignancy, with approximately 30% of patients presenting with metastatic disease at diagnosis, leading to poor prognosis with 5-year survival rate less than 48% [1–3]. Despite the efficacy of antiangiogenic agents targeting the vascular endothelial growth factor (VEGF) pathway [4–6], most patients eventually progress.

In recent years, immunotherapy, including immune-checkpoint inhibitors (ICIs), has shown promising results in the treatment of mRCC [7–9]. While ICIs have demonstrated significant anti-tumor potential, the mechanisms of immune escape in mRCC are complex, and blocking immune-checkpoints alone can have limited efficacy [10, 11]. In addition, ICIs can sometimes induce serious side effects which either cause ceasing of the treatment or death of patients. Therefore, alternative immunotherapeutic approaches are necessary to be explored.

In addition to ICIs, adoptive cell therapies (ACTs), such as chimeric antigen receptor modified T (CAR-T) cells, have shown extraordinary results in hematological malignancies. The data from recent clinical trials showed that CD19 CAR-T therapy in relapsed/refractory large B-cell lymphoma (LBCL) achieved 52–93% objective response rates (ORR) and long-term disease control [12, 13]. And the ELIANA study treating patients with B-cell acute lymphoblastic leukemia showed that CD19 CAR-T therapy was associated with an 81% ORR and durable complete remissions (CR) [14]. Although CAR-T therapies achieved tremendous success in hematological malignancy, their efficacy against solid tumors has been limited due to the absence of tumor-specific antigens [15]. Regarding to tumor-specific immune response, tumor-infiltrating lymphocytes (TILs) have shown clinical efficacy in solid tumors [16, 17], but preparation of TIL requires sufficient tumor tissue, limiting their general applicability [18, 19]. Therefore, for most cancer patients, non-specific ACTs offer a realistic and accessible option.

In essence, T cell-based ACTs provide functional T cells to eliminate malignant cells and have a broad application in cancer patients especially those not suitable for ICIs. The RetroNectin activate killer (RAK**)** cells applied in this study were derived from autologous peripheral blood mononuclear cells (PBMCs) induced by RetroNectin together with anti-CD3 monoclonal antibody and Interleukin-2 (IL-2) ex vivo. They are a heterogeneous population of cytotoxic effector cells, mainly composed of CD8^+^ T and natural killer T (NKT) cells, which obtained a broad spectrum of high anti-tumor activity. Autologous RAK cell immunotherapy was safe and had clinical activity in most cases of solid tumor in our previous clinical observations [20]. Although RAK cells had been implemented in clinic for many years, the dosage-related safety and efficacy remain largely unknown, making further investigation necessary.

In this study, we performed a phase I trial of autologous RAK cell immunotherapy in patients with unresectable metastatic RCC after systemic therapy failure. The trial employed a 3 + 3 dose-escalation design with the primary endpoint of safety and secondary endpoints of objective response rate (ORR) and overall survival (OS). In addition to safety and efficacy, we analyzed changes in peripheral lymphocyte subsets and cytokines after RAK cells infusion.

## Methods

### Study design

This single-center, single-arm phase I clinical trial was designed to assess the safety and efficacy of autologous RAK cells monotherapy as a treatment for patients with unresectable mRCC who had failed at least one line of previous systemic therapy. The trial followed a 3 + 3 dose-escalation design and was carried out in compliance with the National Medical Products Administration (NMPA) and International Conference on Harmonization Good Clinical Practice Guidelines. Ethical approval was obtained from the Cancer Hospital of Chinese Academy of Medical Sciences Ethics Committee (16-056/1135) and was conducted in accordance with the Declaration of Helsinki. Written informed consent was obtained from all patients, who were compensated for travel expenses per visit. The trial was registered with the Chinese Clinical Trial Registry (registration number: ChiCTR1900021334).

**Fig. 1:**
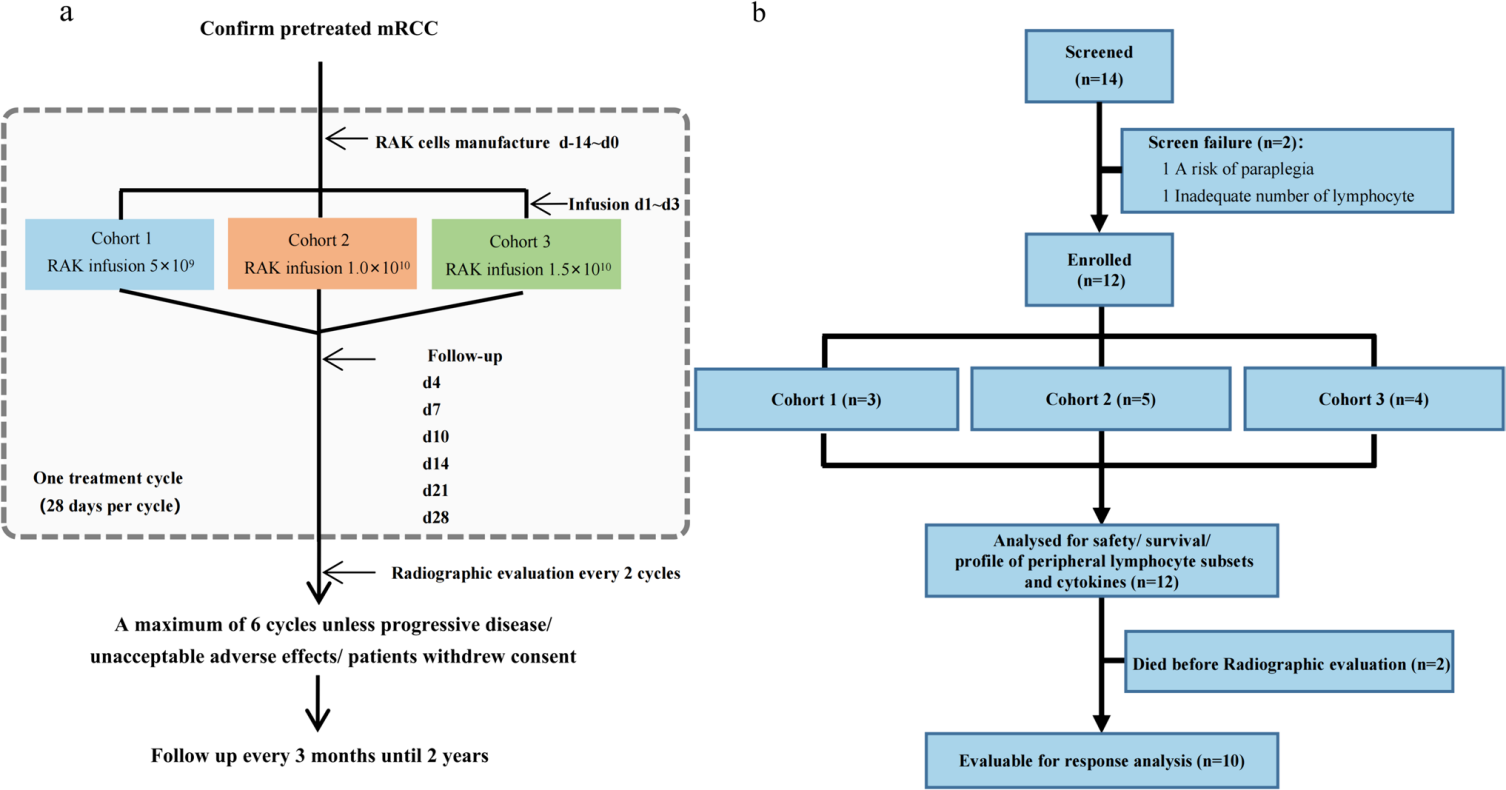
RAK protocol design and consort diagram: **a** protocol schema for RAK cells manufacture, treatment with RAK cells and follow-up and **b** consort diagram indicating the number of patients screened, enrolled in the study and infused with RAK cells

The trial consisted of three dose-level cohorts, with three to six patients enrolled sequentially at doses of 5 × 10^9^, 1 × 10^10^ or 1.5 × 10^10^. Dose escalation was initiated after at least three patients had completed the safety evaluation period (28 days) at a given dose level, and dose-limiting toxicity (DLT) was observed in less than one-third of patients. RAK cells were administered as an intravenous infusion over 3 consecutive days every 28 days as part of a treatment cycle (Fig. 1a). Patients received inpatient clinical monitoring for a minimum of 24 h after each RAK cell infusion. Responses were evaluated using thoracic-abdominal-pelvic contrast-enhanced computed tomography (CT) scans after every two treatment cycles. Patients received up to a maximum of six cycles unless unacceptable adverse effects, progressive disease or withdrawal of consent occurred. In cases patients expressed a strong desire for RAK treatment after study closure, compassionate treatment was permitted, and the dose and cycles of RAK cells were determined by the investigators. Four patients (01002, 01003, 01005 and 01007) received compassionate treatment (supplementary Fig. 1).

### Patients

Eligible patients had documented mRCC; an age of ≥ 18 and ≤ 70 years; a life expectancy of 3 months or more; an Eastern Cooperative Oncology Group performance status (ECOG PS) of 0 or 1; measurable disease according to Response Evaluation Criteria in Solid Tumors (RECIST), version 1.1; a history of at least one line systemic treatment; adequate hematologic and organ function. Patients with a history of active or severe autoimmune disease; conditions requiring the chronic use of systemic immunosuppressants; prior therapy with adoptive cell therapies (ACTs) (e.g., dendritic cell (DC)-CIK or CIK cells, autologous RAK/LAK cells or other ACTs) and chronic infection (e.g., human immunodeficiency virus infection, syphilis and hepatitis B or C) were excluded (supplementary methods).

### Autologous RAK cells manufacture

The manufacture of RAK cells and the quality control experiments were performed by Everbright Inc. Briefly, peripheral blood mononuclear cells (PBMCs) were extracted from 80 ml of peripheral blood from patients. PBMC was expanded ex vivo using RetroNectin together with anti-CD3 mAb and Interleukin-2 (IL-2) for 14 ± 2 days. Final cell products were released after quality control experiments. The eligible cell product should meet the criteria for phenotypic analysis, guaranteed to test negative for mycoplasma, bacteria and fungi, and the endotoxin level was < 0.25 EU.

### Flow cytometry of RAK cell products and lymphocytes

Multiparameter flow cytometry was performed to identify the phenotype of RAK cell products and analyze peripheral lymphocyte subsets. Peripheral blood samples were collected at baseline and after RAK infusion on days 7, 14, 21 and 28 in first two cycles. PBMCs were extracted by Ficoll density gradient separation and cryopreserved. Single-cell suspensions were made by GT-T551 medium with FBS. Single-cell suspensions was stained in PBS with mouse-anti-human antibodies against CD3, CD4, CD8, CD56, CD16, CD25, CD127, CD45RA and CD45RO. Flow cytometry was performed on BD FACSCanto™ II, and data were analyzed by BD FACSDiva™ Software version 9.2.

### Serum cytokine analysis

Serum samples collected before and after RAK infusion on days 7, 14, 21 and 28. The level of IL-2, IL-4, IL-6, IL-10, IFN-*γ* and TNF-α in serum samples was analyzed using cytometric bead array (CBA). The level of TGF-β1, IL-1β and IL-12p70 was measured using an enzyme-linked immunosorbent assay (ELISA). All analysis is entrusted to the Okhealthcare Clinical Medical Institute.

### Outcomes and statistical analysis

The primary endpoint was to assess the safety during this trial and after the treatment ended according to Common Terminology Criteria for Adverse Events (CTCAE) version 4.03. The secondary endpoint was to assess the efficacy of RAK cells every two cycles by RECIST criteria (version 1.1). Thoracic-abdominal-pelvic contrast-enhanced CT was performed at baseline and after every two treatment cycles.

Data on all 12 patients treated were used for summaries of baseline characteristics and adverse events which summarized descriptively by cohort and combined. DLT was defined as one or more of the following related events occurring within 28 days of the first cycle after RAK infusion. Hematologic toxicity: Grade 4 hematologic toxicity ≥ 7 days; Grade 3 neutropenia with fever (≥ 38.5 °C); Grade 3 thrombocytopenia with bleeding; or nonhematologic: including immune-related adverse events, except nausea, vomiting and alopecia. Best of response analysis included the 10 patients who had available radiological examination. Objective responses were confirmed by at least one sequential tumor assessment, and ORR was calculated as [(complete responses + partial responses)/number of patients] × 100, disease control rate (DCR) was calculated as [(complete responses + partial responses + stable disease)/number of patients] × 100. OS and progression-free survival (PFS) were calculated using the Kaplan–Meier method, and 95% confidence interval was included for medians and curves. Statistical analyses were performed using Prism8 GraphPad (GraphPad Software) and R language v.4.2.2.

## Results

### Patient selection and characteristics

Fourteen patients were screened between October 25, 2017, and June 17, 2020. Of these subjects, 12 were consented and received planned protocol therapy. One failed because of a high risk of paraplegia with lumbar metastases disease. Another one failed because of ineligible absolute counts of peripheral blood lymphocyte (Fig. 1a, b).

Clinical characteristics of the 12 patients infused with autologous RAK cells are summarized in Table 1. Patients were predominantly male (75%), with a median age of 57.5 years (interquartile range (IQR), 51.75–62.75 years, range 39–66 years). All patients (100%) had an ECOG PS score ≤ 1. All patients (100%) were initially diagnosed as either localized or locally advanced RCC, performed radical nephrectomy and diagnosed as clear cell RCC by postoperative pathological analysis. All patients (100%) experienced relapse or metastasis afterward. Eleven patients (92%) received one prior antiangiogenic drugs, and 1 patient (8%) received two prior antiangiogenic drugs. Seven patients (58%) belonged to intermediate risk prognostic groups according to the International Metastatic Renal Cell Carcinoma Database Consortium (IMDC), while 5 patients (42%) belonged to favorable groups.

**Table 1:** Clinical characteristics of the mRCC patients

ID	Baseline characteristics before received RAK					Previous surgery history			Previous systemic therapies history
	Age	Gender	ECOG PS	Sites of metastatic disease	IMDC risk group	Pathological stage	Fuhrman nuclear grade	Time from diagnosis to metastatic disease (months)	Drugs
01001	50	F	0	Bo(m), LN(m)	Intermediate	TxN0	III	99	Sunitinib
01002	54	M	1	Lu(m), LN, Bo(m)	Intermediate	T3N0	II–III	8	Sunitinib
01003	64	M	0	Lu(m), LN(m), Ad	Intermediate	T4N0	III–IV	94	Sorafenib
01004	59	M	0	Lu(m), Bo(m), LN(m), Pl(m)	Intermediate	T1N0	II	84	Sorafenib, anlotinib
01005	59	M	0	Lu(m), Bo(m)	Favorable	T1N0	II–III	37	Sorafenib
01006	59	F	0	Lu, PT, AW, LN(m)	Favorable	T1N0	III	19	Sunitinib
01007	56	M	0	Lu, LN(m), Pl(m), Ki	Favorable	T1N1	II	49	Sunitinib
01008	39	M	1	Lu(m), LN(m), PT, Ur	Intermediate	T2N0	III	4	Sorafenib
01009	66	M	1	Lu(m), Pl(m), LN(m), Sc, Bo(m)	Intermediate	T3N1	III	38	Sorafenib
01010	51	M	0	Lu(m), LN(m)	Favorable	T1N0	IV	13	Sorafenib
01011	66	F	0	Lu(m), Ad, LN, PT	Intermediate	T1N0	II	72	Sunitinib
01012	56	M	1	Lu(m), Bo(m), LN(m), Sc, Ki	Favorable	T1N0	NA	98	Sorafenib

Bo, bone; LN, lymph nodes; m, multiple; Lu, lung; PL, pleural; Sc: Subcutaneous; PT: peritoneal; AW: abdominal wall; Ki: Kidney; Ur: ureter; NA: unavailable; IMDC: International Metastatic Renal Cell Carcinoma Database Consortium

### Characteristics of RAK cells

**Fig. 2:**
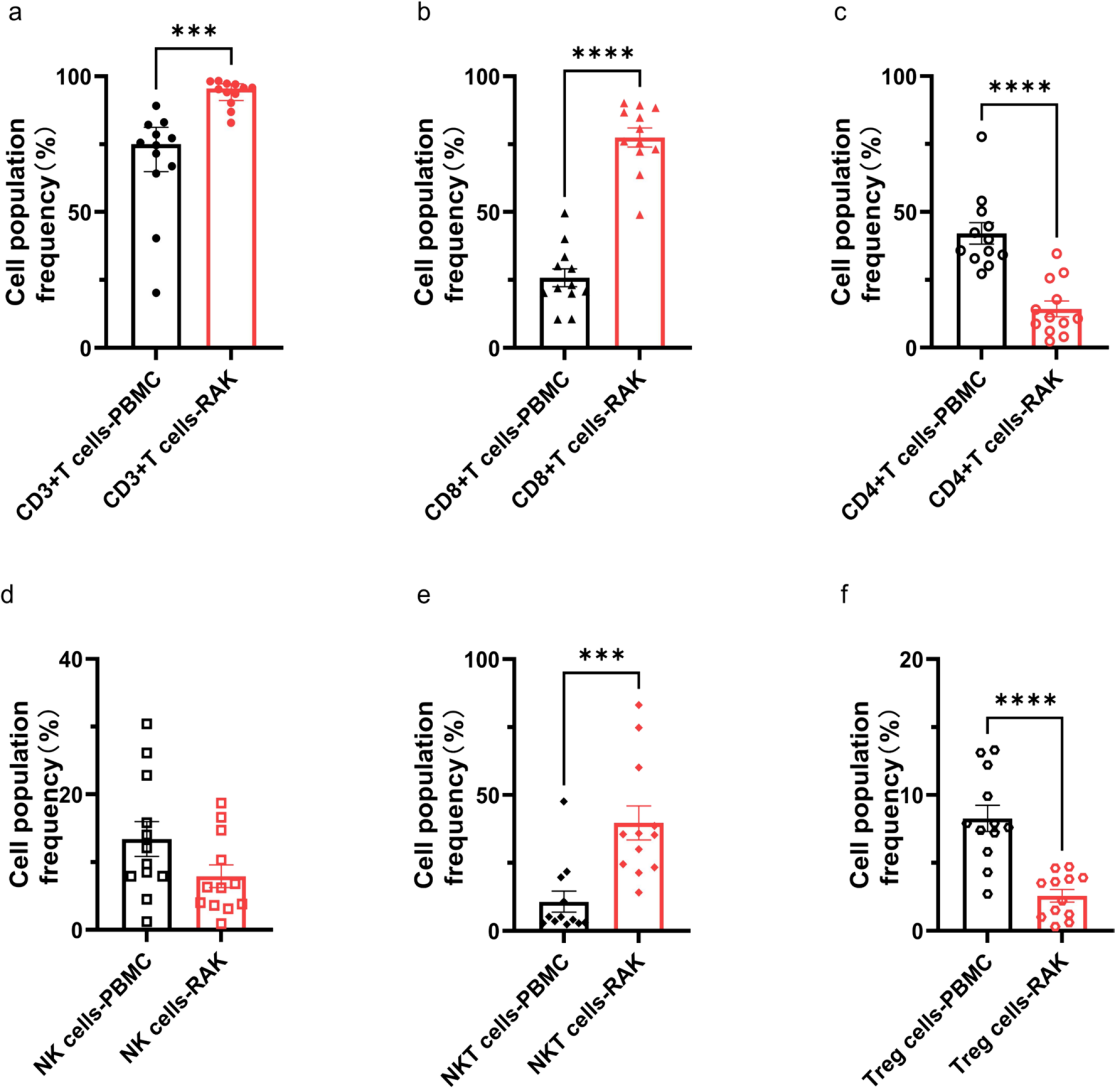
Phenotype of peripheral blood mononuclear cells (PBMCs) and RAK cells. Cell population frequency of **a** CD3 ^+^ T cells (CD3 ^+ ^phenotype), **b** CD8 ^+^ T cells (CD3 ^+^ CD8 ^+^ phenotype), **c** CD4 ^+^ T cells (CD3 ^+^ CD4^ + ^phenotype), **d** NK cells (CD3^-^CD16 ^+ ^CD56^ +^ phenotype), **e** NKT cells (CD3^ +^ CD16^ + ^CD56 ^+^ phenotype) and **f** Treg cells (CD25 ^+^ CD4 ^+^ CD127^low^phenotype) in PBMC and autologous RAK cell product is depicted. Error bars depict s.e.m. *P* values were calculated using a two-tailed t-test

The recourse of RAK cells was derived from autologous peripheral blood cells. The median time of manufacturing was 14 days (range 14–16 days). The PBMC fraction of T cells, CD8^+^ T cells, CD4^+^ T cells, NK cells, NKT cells or Treg cells in each patient was heterogeneous. The frequencies of CD3^+^, CD3^+^CD8^+^, CD3^+^CD4^+^, CD3^−^CD56^+^CD16^+^, CD3^+^CD56^+^CD16^+^ and CD25^+^CD4^+^CD127^low^ phenotype (mean ± SEM) were 68.6 ± 5.7%, 25.8 ± 3.3%, 42.1 ± 4.0%, 13.4 ± 2.6%, 10.7 ± 3.9% and 8.3± 1.0%, respectively. After the stimulation and expansion in vitro, the infusion products from all patients displayed a significant increase in frequencies of total T cells, CD8 ^+^ T cells and NKT cells. The frequency of CD3^+^ T cells, CD8^+^ T cells, NKT cells (mean ± SEM) was 93.8 ± 1.4%, 77.5 ± 3.5% and 39.7 ± 6.3%, respectively, while the frequencies of CD4^+^ T cells (14.4 ± 2.9) and Treg cells (2.6 ± 0.5) were reduced. The manufacture did not affect frequency of NK cells which were 7.9 ± 1.7% afterward (Fig. 2).

### Autologous RAK cells were generally safe in patients

In total, eight patients completed two, one patient (01001) completed four and three patients (01003, 01007, 01012) completed six treatment cycles, respectively. Four patients (01005, 01002, 01003, 01007) received compassionate treatment with RAK (Supplementary Fig. 1). Patients in different cohort received protocol-specified dose of autologous RAK cells. No dose-related toxicity and predefined dose-limiting toxicities (DLTs) within 28 days after the first RAK infusion were observed. Treatment-emergent adverse events (TEAEs) were observed in 11 (91.7%) of 12 patients, among whom 4 patients (33.3%) had TEAE above grade 3 (Table 2). Treatment-related adverse events (TRAEs) were observed in 6 patients (50.0%), the most common symptoms were fatigue (16.7%), rash (16.7%) and increased aspartic transaminase (16.7%), which self-resolved without intervention. Grade 3–5 TRAEs were observed in 2 patients (16.7%). One showed grade 3 hypertension in cohort 1, and the other showed grade 5 myocardial infarction in cohort 3 (Supplementary Table 1).

**Table 2:** Treatment-related adverse events reported with autologous RAK cells

	Cohort 1 (*n* = 3)		Cohort 2 (*n* = 5)		Cohort 3 (*n* = 4)	
	Grade 1–2	≥ Grade 3	Grade 1–2	≥ Grade 3	Grade 1–2	≥ Grade 3
Hypertension	0 (0%)	1 (33%)	0 (0%)	0 (0%)	0 (0%)	0 (0%)
Arrhythmia	1 (33%)	0 (0%)	0 (0%)	0 (0%)	0 (0%)	0 (0%)
Acute myocardial infarction	0 (0%)	0 (0%)	0 (0%)	0 (0%)	0 (0%)	1 (25%)
Increased alanine aminotransferase	0 (0%)	0 (0%)	1 (20%)	0 (0%)	0 (0%)	0 (0%)
Increased aspartic transaminase	0 (0%)	0 (0%)	1 (20%)	0 (0%)	1 (25%)	0 (0%)
Increased γ-glutamyltransferase	0 (0%)	0 (0%)	1 (20%)	0 (0%)	0 (0%)	0 (0%)
Increased alkaline phosphatase	0 (0%)	0 (0%)	1 (20%)	0 (0%)	0 (0%)	0 (0%)
Hypertriglyceridaemia	0 (0%)	0 (0%)	1 (20%)	0 (0%)	0 (0%)	0 (0%)
Fatigue	0 (0%)	0 (0%)	2 (40%)	0 (0%)	0 (0%)	0 (0%)
Rash	0 (0%)	0 (0%)	1 (20%)	0 (0%)	1 (25%)	0 (0%)

In Cohort 1, three patients received a dose of 5 × 10^9^ cells. TRAE was reported in one patient (01003) who developed grade 1–3 transient elevated blood pressure in 2–6 cycles of infusion. Hypertension occurred on the day of infusion and once accompanied with grade 1 arrhythmia. The hypertension fully resolved within 24 h following administration of antihypertensive drugs. In another patient (01002), thrombocytopenia appeared after 28 days of the first cycle of treatment and lasted 150 days. Continue decrease of platelet counts from grade 2 to grade 3 led to discontinuation of RAK therapy after two cycles. Marrow cytology revealed normal counts of marrow megakaryocytes without dysmaturity. The patient received a compassionate treatment with RAK cells afterward when thrombocytopenia alleviated by thrombopoietin receptor agonist. No AEs occurred in compassionate treatment including thrombocytopenia, indicating that thrombocytopenia was unlikely associated with RAK cells therapy.

In Cohort 2, five patients received a dose of 1.0 × 10^10^ cells. TRAEs were reported in 3 patients (01006, 01010, 01012) and no TRAEs above grade 3 were observed. One patient (01012) postponed RAK infusion due to cholelithiasis which has no relation to RAK infusion. Patient 01004 withdrew from RAK therapy because of deteriorated performance status after an pathologic fracture of the right femur.

In Cohort 3, four patients received 1.5 × 10^10^ cell dose. Two patients experienced TRAEs and one of whom (01009) suffered serious AEs (SAEs). Patient 01009 was a 66-year-old man with a history of heavy smoking and hypertension, who had multiple metastasis in the lung, lymph nodes, pleura, skin and bone. At day 22 of first treatment cycle, he had a sudden-onset weakness of the left limbs. Arteriovascular ultrasound suggested severe stenosis of left and right internal carotid arteries and right vertebral artery. Brain MRI scan indicated multiple cerebral infarction lesions. Since the cerebral ischemic stroke was considered to be unrelated to RAK therapy, the patient received another treatment of RAK after symptoms improved with medication. The patient died of an acute myocardial infarction the next day of second RAK infusion. The influence of RAK infusion on patient death could not be excluded.

### Therapy response and efficacy of RAK cells

**Fig. 3:**
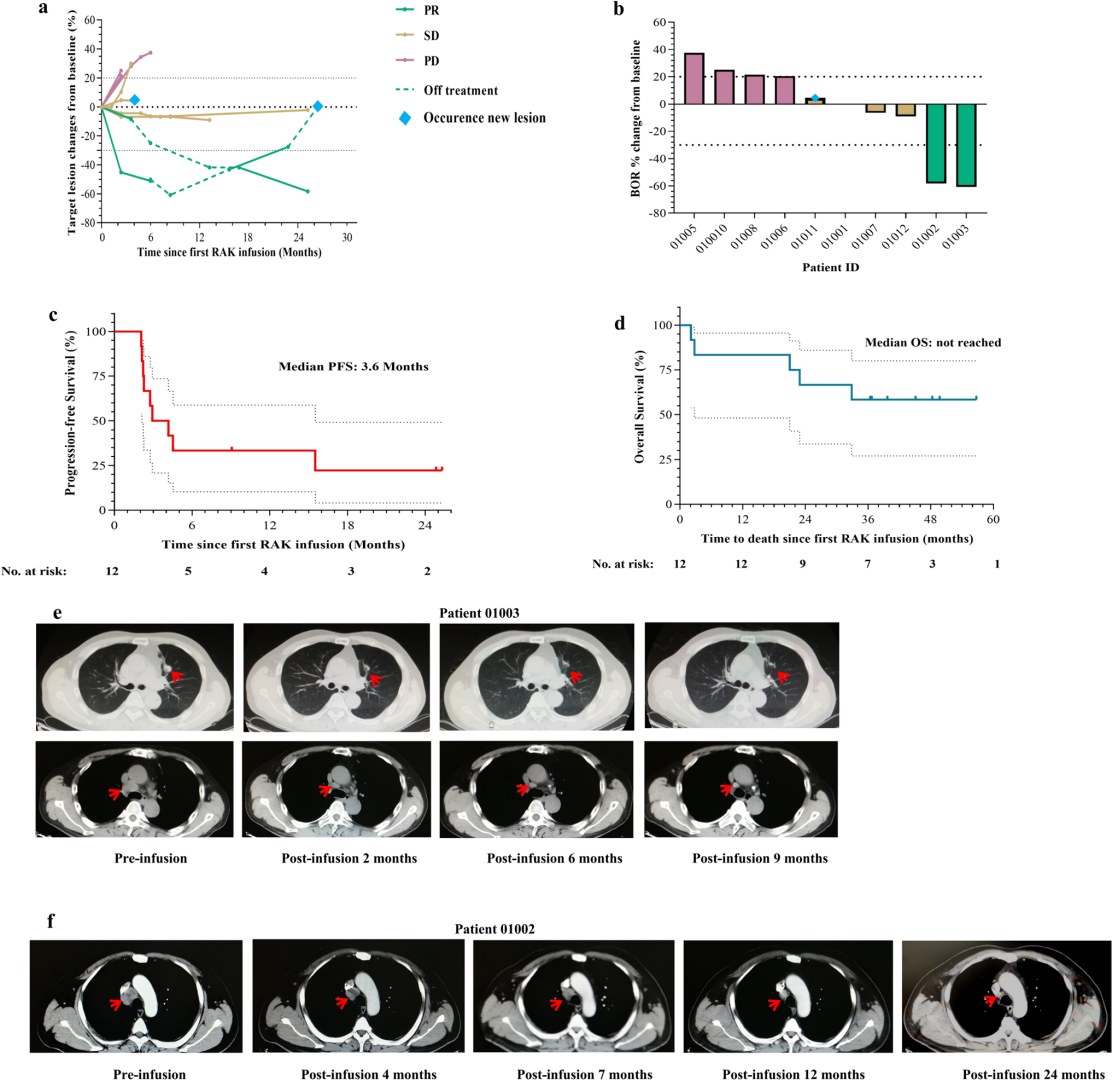
Clinical efficacy of RAK therapy and patient survival. **a** Change in sum of tumor diameters, relative to RAK pre-infusion. None valuable patients (01004 and 01009) are not shown. **b** Waterfall plot showing best overall change in sum of diameter of tumor lesions, relative to RAK pre-infusion. None valuable patients (01004 and 01009) are not shown. Progression-free survival (**c**) and overall survival (**d**) of all treated patients from date of RAK infusion (*n* = 12). Hashtags denote 95% confidence intervals. **e** Tumor lesion of the Lung and mediastinal in patient 01003 shrank after RAK infusion as visualized by CT scan. **f** Tumor lesion of lymph node stations 4R in patient 01002 shrank after RAK infusion as visualized by CT scan

Radiological evaluation by CT scan was performed in 10 of 12 patients, four patients showed tumor regression including two patients achieving a partial response (PR) (Fig. 3a, b). In these four patients, tumor regression was sustained for ≥ 1 years (Fig. 3a). The ORR and DCR were 16.7 and 41.7% for all patients. The median PFS was 3.6 months (95% CI, 2.3, NR) for all patients, and the median OS was not reached (95% CI, 22.9, NR). OS rate at 3 years was 50.0% (95% CI, 21.1, 78.9). There was no dose–response relationship being observed (Fig. 3c, d; Table 3).

**Table 3:** Summary of efficacy results

Variable	Cohort 1 (*n* = 3)	Cohort 2 (*n* = 5)	Cohort 3 (*n* = 4)	All (*n* = 12)
*Best overall response*				
CR, *n*	0	0	0	0
PR, *n*	2	0	0	2
SD, *n*	1	1	1	3
PD, *n*	0	4	3	7
Not evaluable, *n*	0	1^a^	1^a^	2^a^
ORR, *n* (%)^b^	1 (33.3)	0 (0)	0 (0)	2 (16.7) [0.2, 38.5]
DCR, *n* (%)^c^	3 (100)	1 (20)	1 (25)	5 (41.7) [15.2, 72.3]
mPFS (months) [95% CI]				3.6 [2.3–NR]
mOS (months) [95% CI]				NR [22.9–NR]
6-month PFS rate (%) [95% CI]				33.3 [2.0–64.6]
3-year OS rate				50.0 [21.1–78.9]

CR, complete response; PR, partial response; SD, stable disease; PD, progressive disease; ORR, objective response rate; DCR, disease control rate; PFS, progression-free survival; OS, overall survival. NR, not reached

^a^Death before disease assessment; ^b^CR plus PR per RECIST (version 1.1) criteria (investigator assessment); ^c^CR plus PR plus SD per RECIST (version 1.1) criteria (investigator assessment)

Notably, two patients showed durable objective responses. Patient 01003 was a 64-year-old man diagnosed with metastatic clear cell renal cell carcinoma who relapsed after priornephrectomy. He had progressive disease after one prior systemic regimen (sorafenib). The patient received six cycles of RAK infusion (5 × 10^9^ cells) every 28 days. Initial tumor regression occurred at the first CT scan performed 2 months after RAK infusion (Fig. 3a, e). He maintained a partial response for 15.5 months before his lung metastases progressed. Patient 01002 was a 54-year-old man who received right nephrectomy and experienced progression on sunitinib. He received two cycles of RAK infusion (5 × 10^9^ cells) and dropped out due to thrombocytopenia. The delayed initial radiological evaluation by CT scan performed 4 months after RAK infusion showed 8.3% reduction in the target lesion. The response remained in the absence of any anti-tumor therapy and showed 41.7% reduction 13 months after first infusion. The patient then received three cycles compassionate treatment with RAK cells alone every 3 months, and the target tumor lesions continued to shrink with 58.3% reduction. The response was still ongoing following the subsequent systemic treatment with axitinib (Fig. 3a, f).

### Recasted profile of peripheral lymphocyte subsets and cytokines

**Fig. 4:**
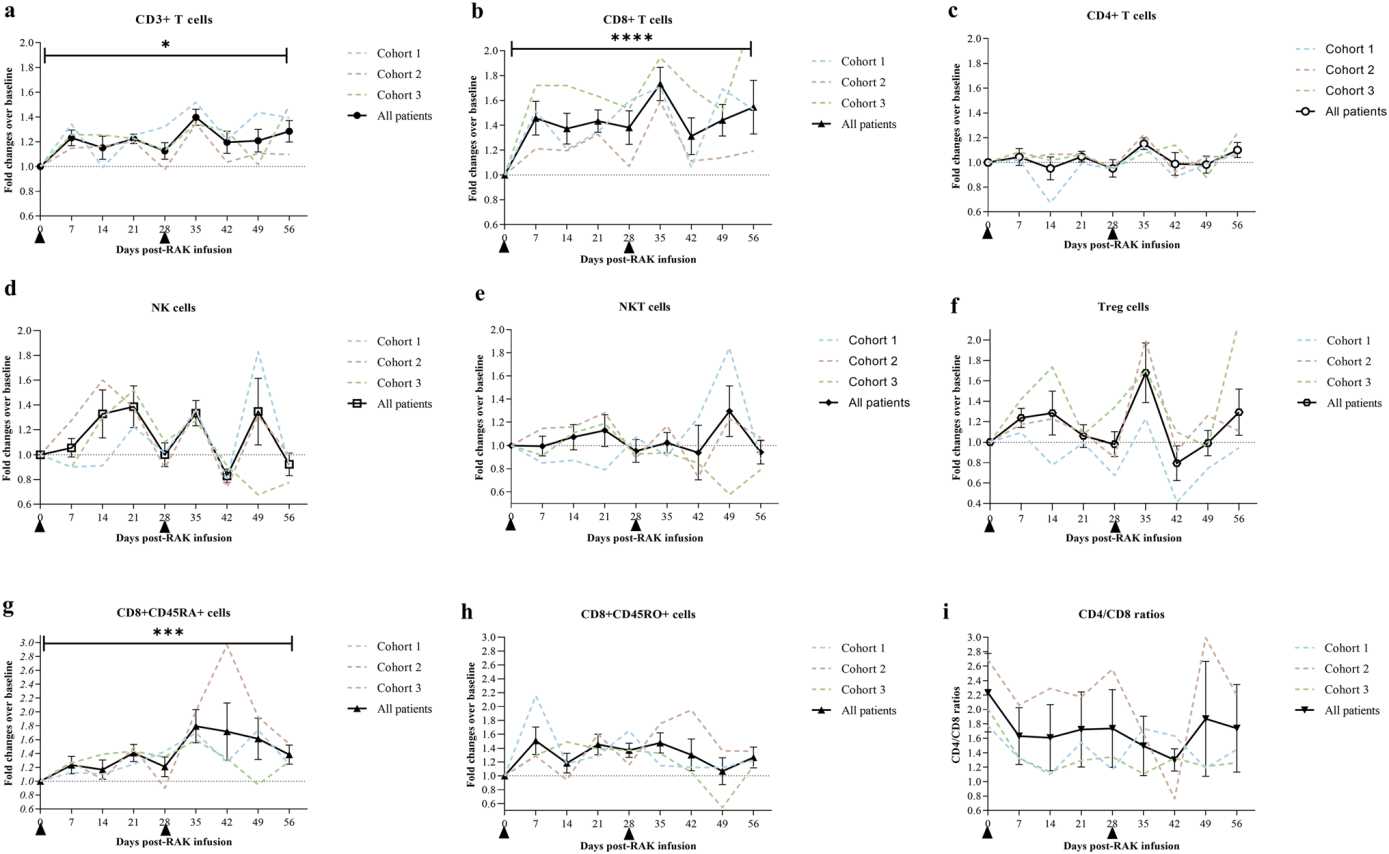
Longitudinal peripheral lymphocyte subsets profiles in mRCC patients treated with RAK cells. Fold changes in peripheral blood absolute counts of **a** total T cells (CD3^+^), **b** CD8^+^ T cells (CD3^+^CD8^+^), **c** CD4^+^ T cells (CD3^+^CD4^+^), **d** NK cells (CD3^−^CD16^+^CD56^+^), **e** NKT cells (CD3^+^CD16^+^CD56^+^), **f** Treg cells (CD4^+^CD25^+^CD127^low^), **g** naive CD8^+^ T cells (CD3^+^CD8^+^CD45RA^+^) and **h** memory and effector CD8^+^ T cells (CD3^+^CD8^+^CD45RO^+^) from baseline (pre-RAK cell infusion) to each time point post-RAK cell infusion were assessed in patients by flow cytometry are depicted as line graphs. (i) The changes in CD4/CD8 ratio in peripheral blood from baseline (pre-RAK cell infusion) to each time point post-RAK cell infusion. Bars denote mean ± s.e.m. of all patients with available time points for analysis (*n* = 12). *P* values were calculated using a two-tailed *t*-test

The effect of RAK infusion on peripheral lymphocyte subsets and cytokines was an exploratory endpoint. All 12 patients were investigated for dynamic changes in the peripheral lymphocyte subset from baseline (day 0) to day 7, day 14 and day 21 post-RAK infusion in first two cycles. There were marked increases in fold change of absolute counts of CD3^+^ and CD3^+^CD8^+^ cells after two cycles of infusion compared with those of the baseline (day 56 vs. Day 0; CD3^+^ *P* = 0.0154; CD3^+^CD8^+^ *P* < 0.0001). Patients in Cohort 3 with 1.5 × 10^10^ cells infusion had higher average peak levels of CD3^+^CD8^+^ cells than those in Cohort 1 with 5.0 × 10^9^ cells infusion and Cohort 2 with 1.0 × 10^10^ cells infusion (Fig. 4, Supplementary Figs. 2, 3). Within the CD8^+^ subset, we found a significant increase in naive CD8^+^ T cells (CD8^+^CD45RA^+^, *P* = 0.0003); while the increase in effector and memory CD8^+^ T cells (CD8^+^CD45RO^+^) with no statistical significance (*P* = 0.176). Additionally, we also found dynamic changes in CD3^+^ and CD3^+^CD8^+^ cells after infusion. We observed an initial increasing phase of CD3^+^ and CD3^+^CD8^+^ cells, peaking on day 7, followed by a decline to day 21, and raised again within 7 days before the infusion thereafter.

**Fig. 5:**
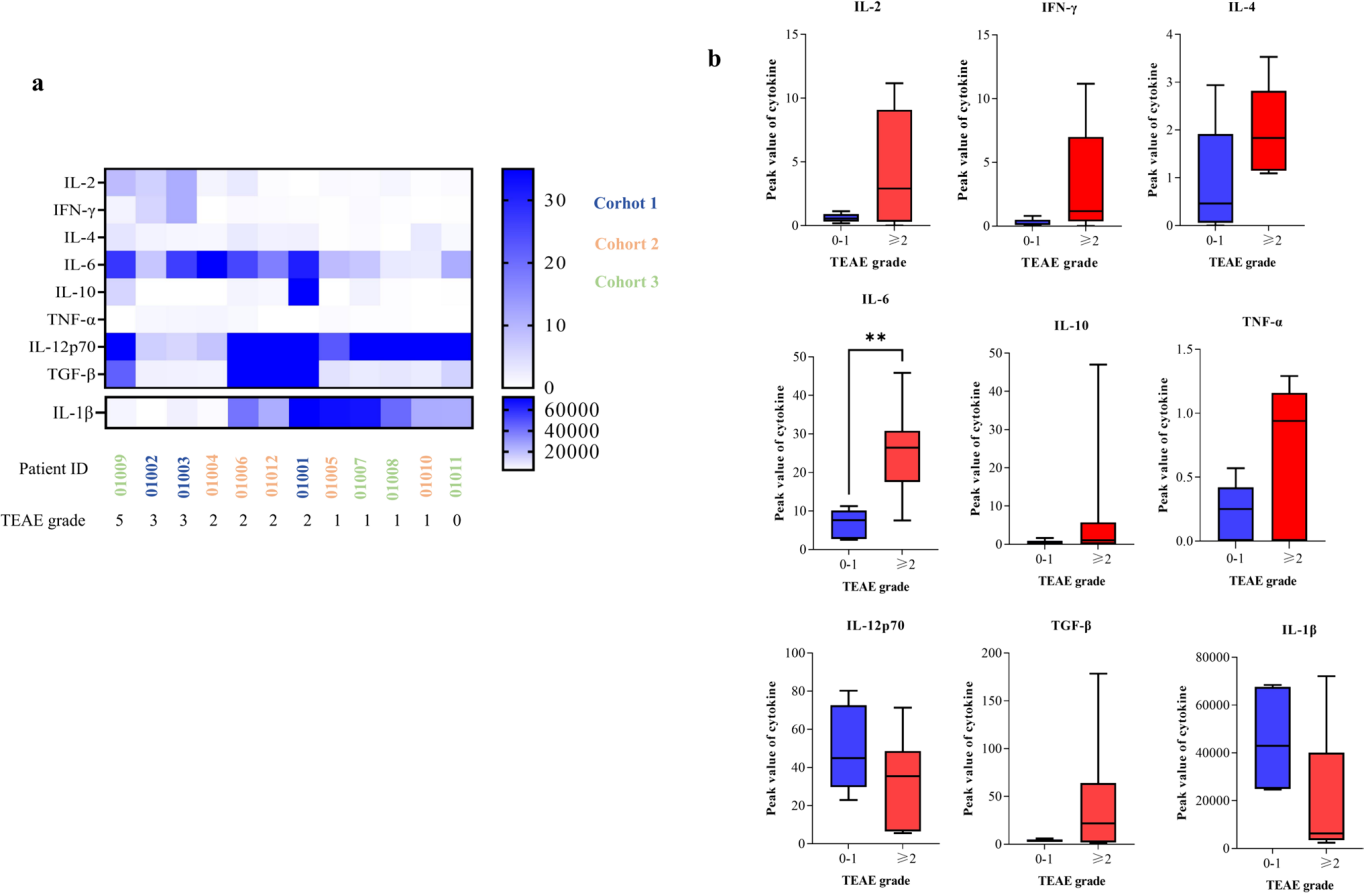
the analysis of peak values of cytokines in peripheral blood: **a** Heat maps and **b** box plots of cytokines peak values after RAK infusion (grouped by TEAE grade, grade ≥ 2, *n* = 7; grade = 0–1, *n* = 5). Box plots were the range of 50% of the central data, with the central line marking median value, whiskers were the range of the data. *P* values were calculated using Mann–Whitney U-test

The relationship among the grade of TEAE, efficacy and the peak values of nine cytokines (IL-2, IFN-γ, IL-4, IL-6, IL-10, TNF-α, IL-12p70, TGF-β and IL-1β) were also examined, respectively. TEAEs above grade 2 were associated with higher peak values of cytokines of IL-2, IL-4, IL-6, TNF-α, TGF-β and a lower value of IL-1β. Among these cytokines, the elevated level of IL-6 was of statistical significance (*P* = 0.006) (Fig. 5). Responders (01003 and 01002) were observed to have higher peak values of IL-2 and IFN-γ after cell infusion compared to those in non-responders.

## Discussion

In this study, the safety and efficacy of autologous RAK cell immunotherapy were evaluated in unresectable metastatic RCC patients who had experienced disease progression after at least one first-line systemic therapy. The autologous RAK cells, mainly composed of CD8 ^+^ T and NKT cells, were successfully prepared for all patients and were well-tolerated with manageable adverse effects. The study showed potential anti-tumor activity with two patients experiencing durable partial responses.

The majority of TRAEs were grade 1 or 2 and ameliorated without intervention, indicating that RAK cell therapy was safe and could be performed on an outpatient basis with minimal supportive care. Treatment-related grade 3–5 toxicity occurred in 2 patients (16.7%), both presented as cardiovascular adverse events. Patient 01003 experienced grade 3 hypertension, and patient 01009 died of acute myocardial infarction. Both patients had a history of hypertension for more than 10 years and heavy smoking for at least 15 years. Notably, patient 01009 had severe atherosclerosis stenosis with plasma hypercoagulable markers (fibrinogen and D-dimer) above the conventional reference range at baseline and in sustained high level during RAK treatment, which indicated that the patient faced high risk of cardiovascular diseases. Furthermore, we observed the enrichment of IL-2, IL-4 and IL-6 in patients who experienced TEAEs above grade 2, especially in patient 01009. It was reported that cytokines, especially IL-6, play a role in immune-related adverse events such as cytokine release syndrome (CRS) [21–23]. And the hyperinflammatory state induced by CAR-T therapy had been reported to increase the risk of thrombosis [24–26] and was involved in the development of various inflammatory cardiac pathologies [27]. In this study, we failed to obtain more evidence to understand the inflammatory state at the time SAE occurred because the patient had been discharged without any symptoms after the second cycle of infusion. But according to the consistent increasing trend of cytokines and the level of CD8^+^ T cells in PBMC (Supplementary Figs. 4, 5) after first cycle of RAK infusion, we cannot exclude the contribution of the infusion to the hyperinflammatory state, which might be one of underlying factors associated with his acute myocardial infarction. To our knowledge, this is one of the first studies reporting acute myocardial infarction associated with non-specific ACT. Although the data provided could not explain the detailed mechanisms, this study, however, alert us the existence of high-risk population for autologous ACT. Our study suggested that people with cardiovascular disease (such as venous or arterial thrombosis, medication history of antiangiogenic agents, etc.) belong to high-risk group for adoptive cell therapy. These findings implied that any future study needs close cardiac monitoring and likely caution in selecting patients. Peripheral arterial and venous examinations and laboratory tests for hypercoagulate diagnosis (fibrinogen, D-dimer and platelet counts) should be included in clinical observation to avoid venous or arterial thromboembolic events. In addition, the volume of 400 ml intravenous fluid was another potential risk factor because of increase of cardiac preload and myocardial oxygen consumption. Therefore, we will optimize the infusion fluid volume down to 100 ml in the future trials (ChiCTR1800019336).

In the study, 4 patients (33.3%) showed tumor shrinkage, two of them achieved durable partial responses. Although this study was not designed to show the efficacy, RAK cells has signal of clinical activity in a subset of patients, which warrant further study. The relative small sample size and inclusion of all patients with more than two metastatic sites limited interpretation of PFS (3.6 months; 95% CI, 2.3, NR) and ORR (16.7%). The delays in treatment due to COVID-19 epidemic might also have had a negative impact on the efficacy. Notably, a higher proportion of patients with IMDC favorable and intermediate risk was included in this study; thus, any efficacy benefit should be confirmed by further large sample size observations. Besides, our study did not include patients who had received prior treatment with ICIs because they were not widely available in China when this phase I trial was conducted. At present, the combination treatments based on ICIs have already been recommended for mRCC. Therefore, the future phase II trial will enroll mRCC patients who have failed previous regimens including the combination therapies with ICIs.

The durability of objective responses in two patients treated with RAK cells was notable, with response sustained for more than 1.5 years in these patients. Patient 01002 experienced treatment break after two cycles of RAK infusion for more than 1 years due to thrombocytopenia. Interestingly, the tumor continued to shrink during this period, which suggested RAK treatment had potential to activate autoimmune responses. In a post hoc exploratory analysis, we assessed the changes of peripheral blood lymphocyte subsets and cytokines at baseline and over a relatively short period after RAK infusion. A transient elevation of CD3^+^, CD3^+^CD8^+^ T cells was observed within 7 days post-infusion followed by a decline, but then, they increased spontaneously in 14–21-day post-infusion. This also implicated that RAK infusion initiates adaptive immune response. It was reported that adoptive cell therapy with naive T cell subsets associated with superior persistence and anti-tumor immunity than with subsets containing more-differentiated effector memory and effector T cells [28, 29]. The analysis of the phenotype change of peripheral T cells in our study revealed that RAK infusion was associated with a greater fold change in levels of naive CD8^+^ T cells. It suggested that the RAK might activate more naive T cell subsets, although we do not have direct evidence to support this hypothesis which need to be further studied. In this study, we did not observe a correlation between the response with level of CD8^+^ T cells or its subsets in peripheral blood. However, in patient 01002 and 01003 we observed high peak values of IL-2 and IFN-γ, the cytokines play important roles in anti-tumor T cell responses in TME [30]. Due to the small sample size and limited varieties of cytokines examined in this study, we did not find other efficacy associated with cytokine analysis.

In this study, the majority of patients experienced stable or progressive disease. In patient 01005, the size of his target tumor lesions were doubling after first two cycles of RAK infusion, while the tumor growth rate reduced significantly during the subsequent compassionate RAK infusion, which implied that RAK treatment requires time to exhibit efficacy. Thus, immediate withdrawal of RAK treatment based on disease progression seemed not to be a proper choice. Patient 01003 who had a durable disease control after six cycles of RAK infusion until experienced disease progression in 9.5 months. Thus, it is possible that RAK treatment should be continued even the disease is under control, but the optimal duration of RAK treatment remains unclear and requires further investigation. We also considered that 28 days as a treatment cycle was more reasonable than 3 months a cycle, because the tumor growth of patient 01003 was out of control in compassionate RAK treatment. The infusion dose of 1.5 × 10^10^ RAK cells is not recommended because its efficacy was not superior to those of either 5 × 10^9^ or 1.0 × 10^10^ dose and might increase risk of cardiovascular events. Patients in low-dose RAK cells cohort (5 × 10^9^) seemed to show higher activity than higher-dose cohorts (1 × 10^10^ and 1.5 × 10^10^), which may be consistent with the point recently made by Thall et al. [31] that some cellular therapies in oncology (e.g., NK cell therapies) show higher efficacy at lower doses. In this case, designs other than conventional 3 + 3 dose escalation, such as a randomized controlled design, should be used for optimal dose-finding of autologous RAK cell immunotherapy in the future.

In conclusion, autologous RAK cell immunotherapy is generally safe with potential evidence of clinical activity in a subset of patients with unresectable mRCC who failed first-line therapy but may increase risk of serious or fatal cardiovascular events, particularly in high-risk patients. The findings in this phase I trial indicated that RAK cells was a potential alternative treatment for patients with mRCC. Future large-size, prospective, randomized investigation is required to further validate RAK therapy, as well as identification of biomarkers to predict patient response.

### Supplementary Information

Below is the link to the electronic supplementary material.

Supplementary file1 (PDF 473 KB)

## Data Availability

Relevant data and analysis are published in this manuscript. Data used during this study are available from the corresponding author on reasonable request.
