# Supplementary material for: A phase I trial of autologous RAK cell immunotherapy in metastatic renal cell carcinoma
Source: Cancer Immunol Immunother. 2024 Apr 20;73(6):107. doi: 10.1007/s00262-024-03680-y (PMC11032301; doi:10.1007/s00262-024-03680-y)
Supplement: Supplementary file 1 — Supplementary file1 (PDF 473 KB) [file 262_2024_3680_MOESM1_ESM.pdf]

Supplementary material:

Supplementary Methods:

Inclusion criteria included:

1.Aged ⩾18 and ⩽70 years, male or female.

2.Historically confirmed mRCC and with disease progression after 1 line systemic treatment.

3.According to Response Evaluation Criteria in Solid Tumors (RECIST) version 1.1, there are measurable lesions.

4.ECOG performance status (PS) score of 0 or 1 within 24 hours before collections for peripheral blood.

5.Estimated life expectancy >3 months.

6.Adequate hematologic reserve within 24 hours before collections for peripheral blood,as defined by absolute neutrophil count >1.5×109/L, lymphocyte count >1.1×109/L, platelet count>80×109/L. no transfusion or cell growth factor was allowed within 1 week before the test.

7.Adequate organ function before enrollment, as defined by the Left ventricular ejection fraction (LVEF) >50% calculated by cardiac ultrasound, no obvious abnormality observed by electrocardiogram, peripheral oxygen saturation (SaO2) >90%, Creatinine clearance (CG formula) >50ml/min, serum total bilirubin <1.5× upper limit of normal (ULN) and serum aspartate transaminase/alanine transaminase <2×ULN.

8.Pulmonary embolism was assessed as normal (Pauda score and risk stratification were used in internal medicine).

9.Men who are fertile or women who are at risk of becoming pregnant must use highly effective contraceptives during the trial(e.g., oral contraceptives, intrauterine contraceptives, abstinence or barrier contraceptives combined with spermicide), and are being treated Continue contraceptive use for 3 months after termination.

10.Sign a written informed consent.

11.Subjects of reproductive potential must agree to use acceptable birth control methods.

Exclusion criteria included:

1.A history of other malignant diseases within the past 5 years.

2.Subjects who require the chronic use of systemic immunosuppressant.

3.History of active or severe autoimmune disease requiring immunosuppressive therapy.

4.Previous use of dendritic cell (DC)-CIK or CIK cells, autologous RAK/LAK cells or other ACT.

5.History of organ transplant.

6.Less than 4 weeks after the completion of previous anti-tumor treatment.

7.Severe or uncontrolled epilepsy, central nervous system diseases, cerebrovascular accidents, heart failure, or other uncontrollable accompanying diseases.

8.Subjects with ongoing or active infection; active hepatitisB, hepatitisC, syphilis or human immunodeficiency virus infection.

9.History of allergy to ingredients of RAK products (human serum albumin and IL-2).

Compassionate treatment:

Four patients received compassionate treatment with RAK (01005, 01002,01003,01007). Patient 01003 received 3 cycles compassionate treatment every 3 months per cycle subsequently after he progressed. Patient 01002 experienced treatment break after 2 cycles of RAK infusion due to thrombocytopenia. He received received 3 cycles compassionate treatment every 3 months per cycle after 16 months of last infusion. Patient 01007 had received compassionate treatment with 3 cycles every 3 months per cycle after he completed 6 cycles of RAK infusion. Patient 01005 received compassionate treatment with 4 cycles every 28 days even though he had progressive disease after 2 cycles of RAK infusion.

Supplementary Figure 1, Clinical follow-up of all 12 patients after RAK infusion in a Swimmer' s plot**.**

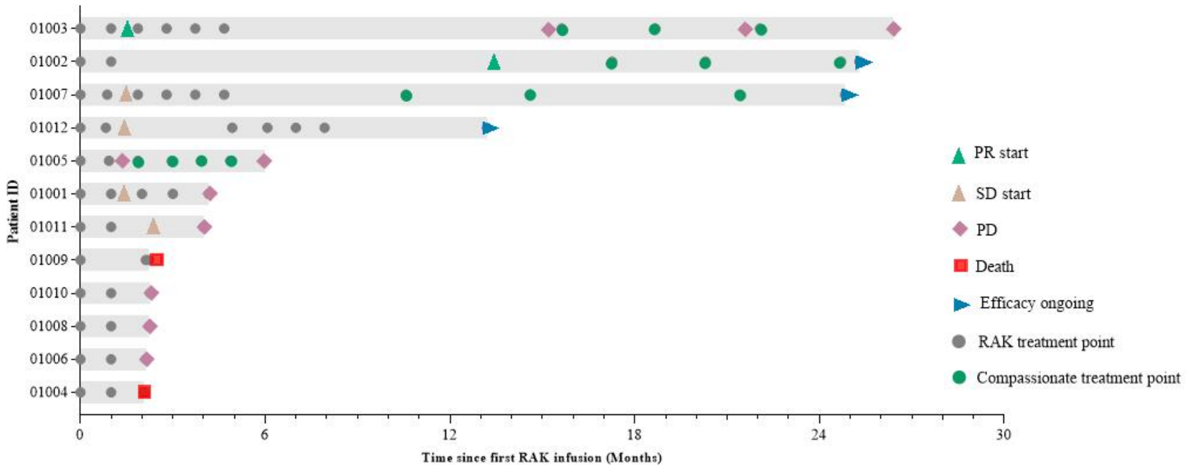

Note: The total infusion cycles for all 12 patients. Eight patients completed 2, 1 patient (01001) completed 4 and 3 patients (01003, 01007, 010012) completed 6 treatment cycles respectively. Four patients (01005, 01002,01003,01007) received compassionate treatment.

Supplementary Figure 2, Longitudinal peripheral lymphocyte subsets profiles in mRCC patients treated with RAK cells.

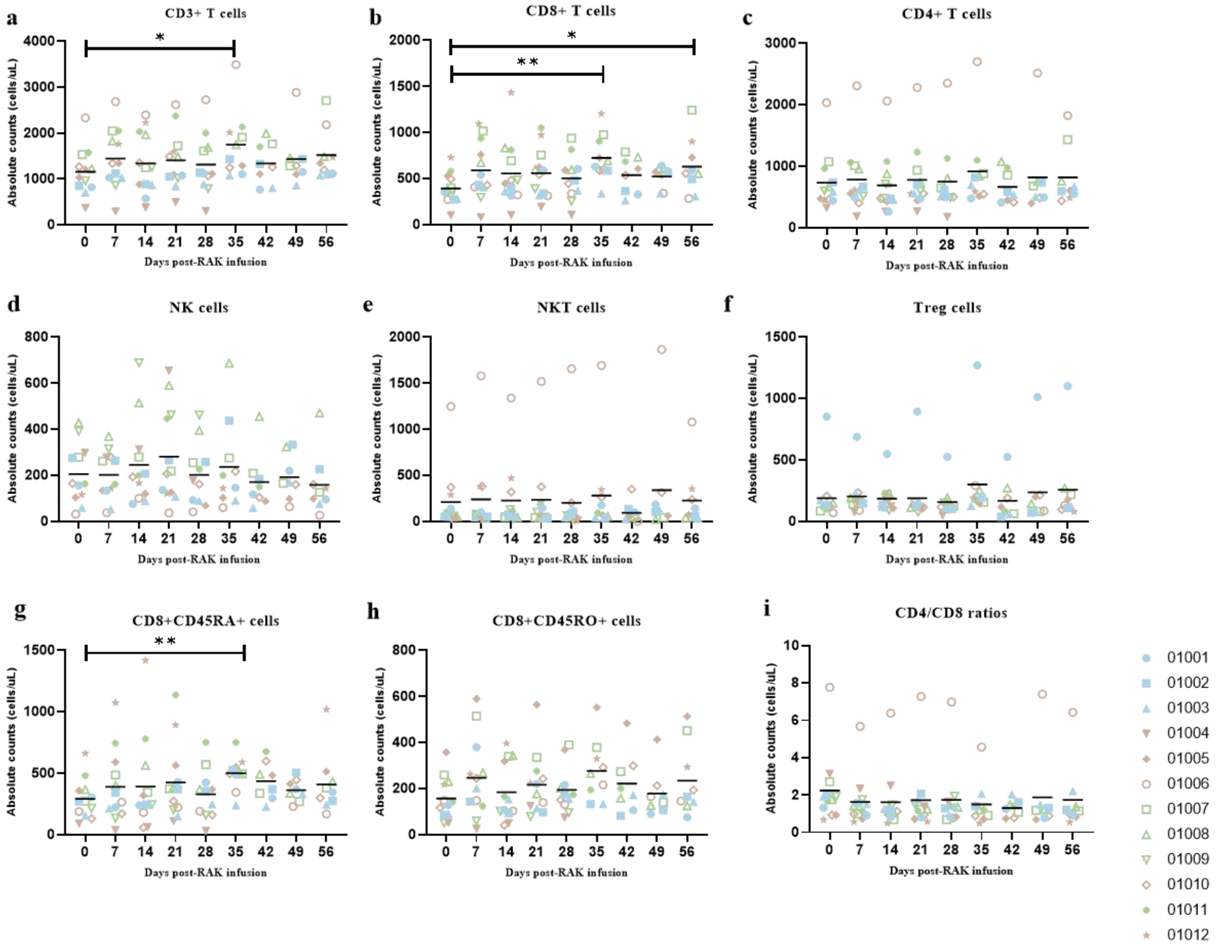

Note: Peripheral blood absolute counts of (a) total T cells ($CD3^{+}$), (b) $CD8^{+}$ T cells ($CD3^{+}CD8^{+}$), (c) $CD4^{+}$ T cells ($CD3^{+}$ $CD4^{+}$), (d) NK cells ($CD3^{-}$ $CD16^{+}CD56^{+}$), (e) NKT cells ($CD3^{+}CD16^{+}CD56^{+}$), (f) Treg cells ($CD4^{+}CD25^{+}CD127^{low}$), (g) naive $CD8^{+}$ T cells ($CD3^{+}CD8^{+}CD45RA^{+}$), (h) memory and effector $CD8^{+}$ T cells ($CD3^{+}CD8^{+}CD45RO^{+}$) from baseline (pre-RAK cell infusion) to each time point post-RAK cell infusion were assessed in patients by flow cytometry are depicted as line graphs. i. The changes in CD4/CD8 ratio in peripheral blood from baseline (pre-RAK cell infusion) to each time point post-RAK cell infusion. Individual data points for each patient and means are exhibited in all panels(n= 12). P values were calculated using a two-tailed t-test. The absolute counts of $CD3^{+}$ cells increased significantly after the second infusion cycle compared with those levels of baseline (day 35 vs. day 0, P=0.037). The absolute counts of $CD3^{+}CD8^{+}$ cells increased significantly after the second infusion cycle compared with those levels of baseline (day 35 vs. day 0, P=0.001; day 56 vs. day 0, P=0.032). The absolute counts of $CD8^{+}CD45RA^{+}$ cells increased significantly after the second infusion cycle compared with those levels of baseline (day 35 vs. day 0, P=0.002).

Supplementary Figure 3, Longitudinal peripheral lymphocyte subsets profiles in mRCC patients treated with RAK cells.

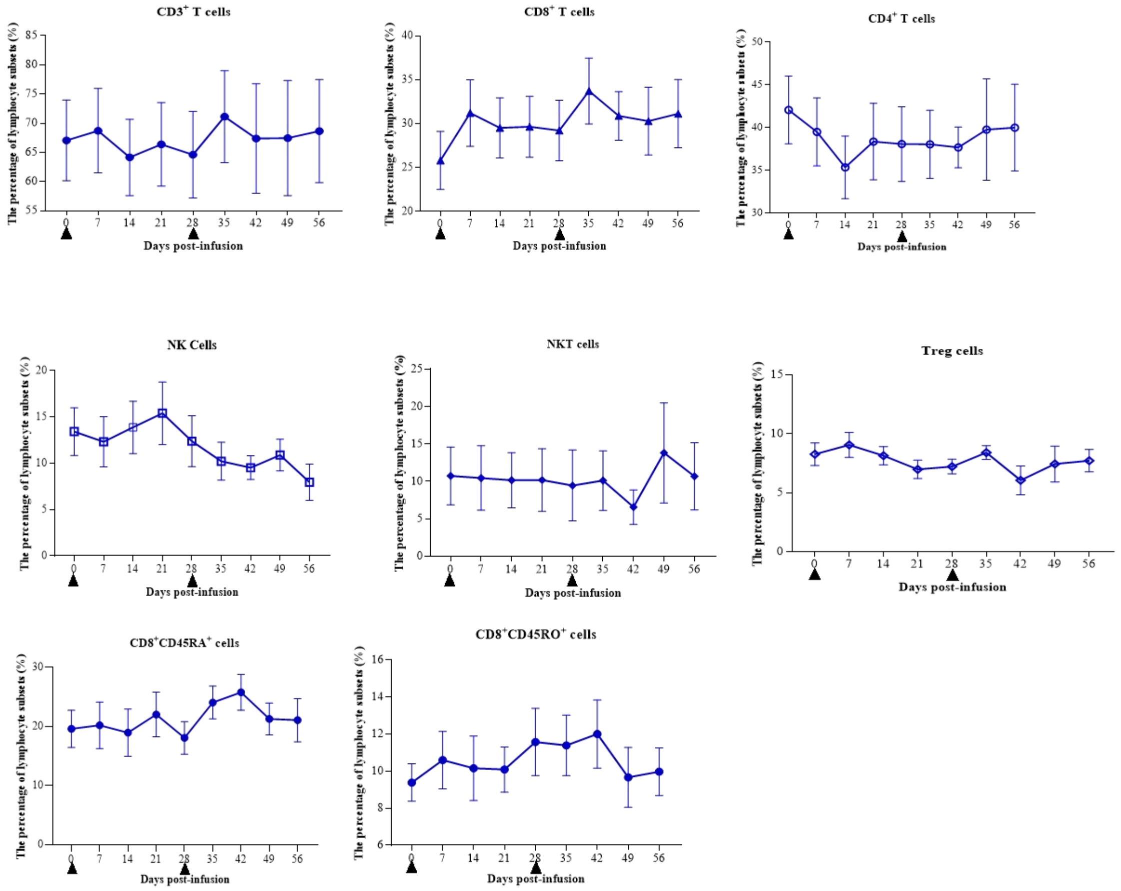

Note: The percentage of peripheral blood lymphocyte subset of (a) total T cells (CD3+), (b) CD8+ T cells (CD3+ CD8+), (c) CD4+ T cells (CD3+ CD4+), (d) NK cells (CD3- CD16+CD56+), (e) NKT cells (CD3+ CD16+CD56+), (f) Treg cells (CD4+CD25+CD127low), (g) naive CD8+ T cells (CD3+CD8+CD45RA+), (h) memory and effector CD8+ T cells (CD3+CD8+CD45RA+) from baseline (pre-RAK cell infusion) to each time point post-RAK cell infusion were assessed in patients by flow cytometry are depicted as line graphs. Bars denote mean ± s.e.m. of all patients with available time points for analysis (n= 12). P values were calculated using a two-tailed t-test.

Supplementary Figure 4, Longitudinal serum cytokines (IL-2, IL-4, IL-6) in peripheral blood of mRCC patients treated with RAK cells.

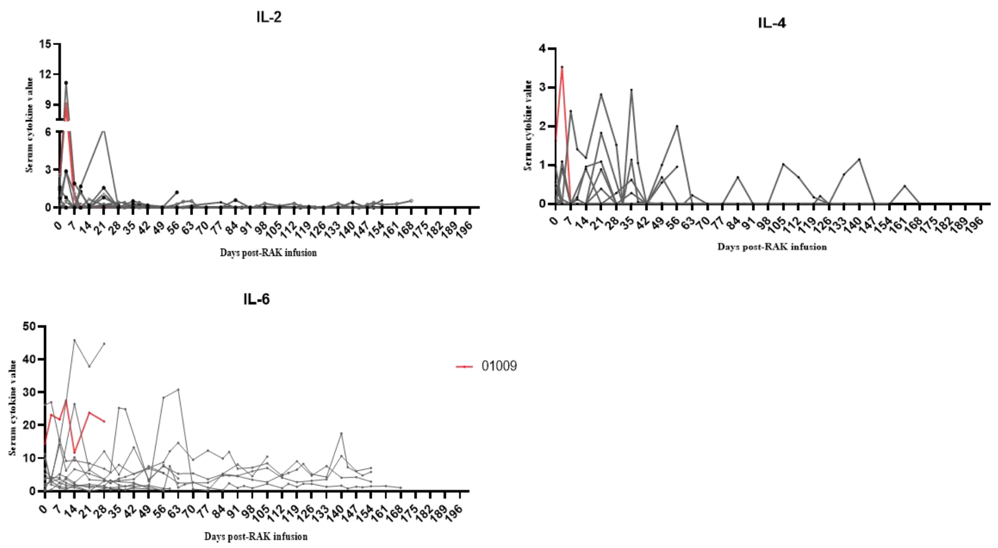

Note: Patient 01009 (red line) exhibited high level of IL-2, IL-4, IL-6 after RAK infusion.

Supplementary Figure 5, Longitudinal measurements of serum IL-6 and CD8+ T cells before and after RAK infusion.

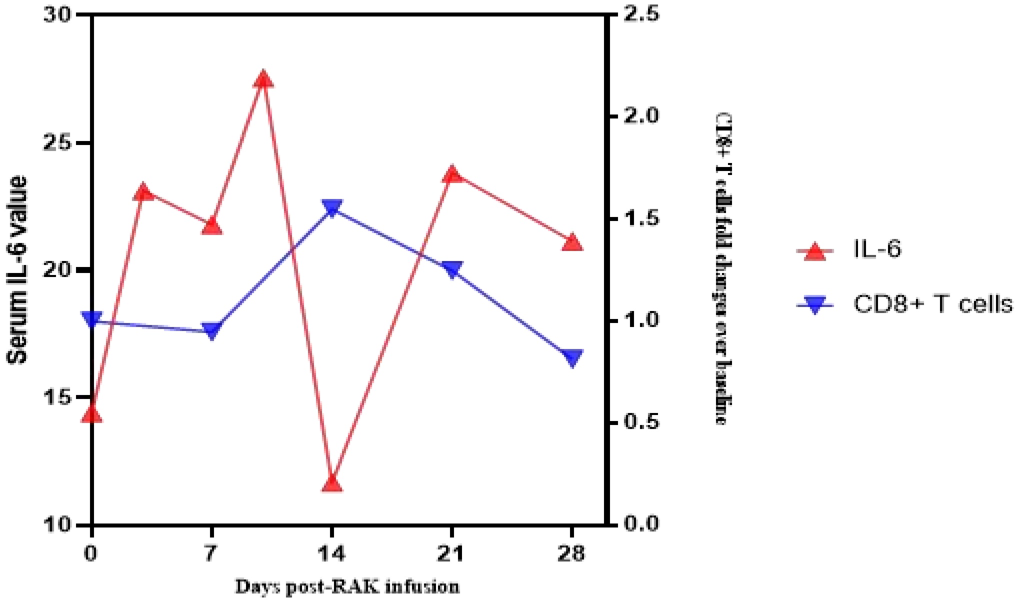

Supplementary Table 1: Treatment-emergence adverse events reported with autologous RAK cells.

Patient ID	Corhot	Correlation with RAK treatment	Adverse event	Highest Grade	Frequency (times)	Longest duration (days)	Time from last infusion to Event days (infusion cycle)
01001	1	Unrelated	Rash	1	1	30	20
		Unlikely	Anemia	2	1	14	28
01002	1	Unlikely	Thrombocytopenia	3	1	150	28(1)
01003	1	probable	Hypertension	3	9	0	0
		possible	Arrhythmia	1	1	0	0
01004	2	Unrelated	Cough	2	1	0	0(2)
		Unrelated	Fracture	2	1	17	1(2)
01005	2	Unrelated	Hypertriglyceridaemia	2	1	90	12(1)
01006	2	possible	Fatigue	1	1	18	3(2)
		possible	Rash	2	1	0	0(1)
		Unrelated	Expectoration	2	1	28→	7(2)
		Unrelated	Cough	2	1	28→	7(2)
		Unrelated	Urinary tract infection	2	1	1	16(2)
01007	3	possible	Rash	1	2	12	10(2);11(1)
		possible	Increased aspartic transaminase	1	1	7	28(6)

		Unrelated	Increased blood cholesterol	1	1	126	11(1)
		Unrelated	Hypertriglyceridaemia	1	1	86	25(3)
		Unrelated	Proteinuria	1	1	18	24(2)
		Unrelated	Hyperuricemia	1	1	86→	25(3)
		Unrelated	Increased blood urea	1	1	1→	28(6)
01008	3	Unrelated	Increased alanine aminotransferase	1	1	14	21(2)
		Unrelated	Increased aspartic transaminase	1	1	14→	21(2)
		Unrelated	Increased blood cholesterol	1	1	27	12(1)
		Unrelated	Increased blood creatinine	1	1	1→	8(3)
		Unrelated	Hyperuricemia	1	1	39→	24(1)
01009	3	Unrelated	Acute cerebral ischemic stroke	2	1	45	22(1)
		Unrelated	Stress ulcer hemorrhage	2	1	45	22(1)
		Unrelated	Hypertension	3	1	1	22(1)
		possible	Acute myocardial infarction	5	1	1	1(2)

01010	2	possible	Fatigue	1	1	17	1(1)
01012	2	possible	Increased alanine aminotransferase	1	1	110	30(1)
		possible	Increased aspartic transaminase	1	1	110	30(1)
		possible	Increased γ-glutamyltransferase	1	2	110	30(1), 10(3)
		possible	Increased alkaline phosphatase	1	1	110	30(1), 10(3)
		possible	Hypertriglyceridaemia	1	2	14	118(2), 10(3)
		Unrelated	Blood bilirubin increased	1	1	13	105(2)
		Unrelated	cholelithiasis	2	1	13	5(3)

→ AE continued to the end of follow-up.
